# Monopolar Transurethral Resection of Prostate for Benign Prostatic Hyperplasia in Patients With and Without Preoperative Urinary Catheterization: A Prospective Comparative Study

**DOI:** 10.7759/cureus.16705

**Published:** 2021-07-28

**Authors:** Baikuntha Adhikari, Anil Shrestha, Robin B Basnet, Parash M Shrestha, Binod B Gharti, Arvind K Shah

**Affiliations:** 1 Urology, National Academy of Medical Sciences, Bir Hospital, Kathmandu, NPL

**Keywords:** acute urinary retention, benign prostatic hyperplasia, monopolar, transurethral resection of prostate, trial without catheter

## Abstract

Background

A significant proportion of patients undergo surgery for benign prostatic hyperplasia following acute urinary retention. Studies have reported conflicting results of improvement following transurethral surgery in these patients.

Objective

To compare perioperative complications and postoperative voiding parameters in patients undergoing monopolar transurethral resection of prostate with and without preoperative Foley catheterization.

Methods

A prospective non-randomized study was conducted in patients undergoing monopolar transurethral resection of prostate for symptomatic benign prostatic hyperplasia. Patients were divided into those with Foley catheterization preoperatively (n=52), and those without catheters (n=90). Change in hemoglobin level, the resected volume of prostate, complications and the need for postoperative catheterization were compared. Postoperative symptoms score using International Prostate Symptom Score, maximum flow rate and post-void residual volume were assessed at three months follow up.

Results

The mean operative duration, length of stay and resected volume were higher in those patients with catheters; however, no significant differences were noted for mean hemoglobin level change and need for postoperative recatheterization. Three patients in each group required recatheterization and, all were catheter-free at one week postoperatively. Complications developed in 16.1% (n=23) with most of them being Clavien I. Patients with catheters had a lower postoperative maximum flow rate than those without it (16.90 vs 19.75 mL/sec). Patients with catheters had a significantly better postoperative quality of life and symptom score.

Conclusion

Monopolar transurethral resection of prostate in patients with preoperative per-urethral Foley catheter for acute urinary retention had similar postoperative voiding parameters with comparable complication rates to those without a catheter.

## Introduction

Benign prostatic hyperplasia (BPH) prevalence increases with age and affects nearly three-quarters of men by the seventh decade of life [1,2]. Acute urinary retention (AUR) is the predominant complaint in a significant proportion of patients (20%-42%) undergoing surgery for BPH [3-5]. Even with the availability of various transurethral procedures, monopolar transurethral resection of prostate (TURP) is still considered the reference standard surgical treatment of BPH for the prostate size of 30-80 mL and continues to be one of the most frequently performed procedures in urological practice [6-10].

Higher incidence of perioperative complications, poor postoperative voiding outcomes and increased postoperative recatheterization rates have been documented in patients undergoing monopolar TURP in patients with a history of AUR [11,12]. In view of these findings, patients with AUR were counseled regarding the higher risk of perioperative morbidity. In contrast, recent studies have shown a reduced complications rate along with the reduced rate of recatheterization with similar voiding parameters postoperatively in these patients [13,14].

In our part of the world, patients have preoperative Foley catheterization for a longer duration due to limited availability of urological care outside the major cities, financial constraints and even fear of surgery, in addition to the limited slots available for surgery in public hospitals [5,15]. There are limited studies addressing both the objective and subjective voiding outcomes of TURP among catheterized patients after failure of at least one trial without catheter (TWOC). Hence, this study was undertaken to compare outcomes of TURP between patients with per-urethral Foley catheter preoperatively after failed TWOC and those with no preoperative Foley catheterization. The outcome of this study can be useful in counseling patients with catheters preoperatively.

## Materials and methods

This prospective comparative study was conducted in patients undergoing TURP for the period of 18 months from March 2019 to August 2020 in the Department of Urology of Bir Hospital, Kathmandu, Nepal. Ethical clearance was obtained from the Institutional Review Board of the National Academy of Medical Sciences (Ref. no. 529/077/78). Informed written consent was obtained from all patients.

Patients requiring TURP were divided into two groups, those with preoperative per-urethral Foley catheter after the failure of at least one TWOC (Group 1) and those with moderate to severe bothersome LUTS but no Foley catheter preoperatively (Group 2). Patients with a history of prostate surgery, prostate cancer, bladder cancer, urethral stricture surgery, the concurrent presence of urinary bladder stone, urethral stricture, neurogenic bladder and the presence of large urinary bladder diverticula were excluded.

Patients were assessed preoperatively performing general physical examination including digital rectal examination and brief neurological examination. The patient’s age, body mass index (BMI), American Society of Anesthesiologists’ classification (ASA) and prostate-specific antigen (PSA) level were recorded. Group 2 underwent preoperative uroflowmetry to document maximum flow rate (Qmax) and flow pattern, together with post-void residual (PVR) volume, and International Prostate Symptom Score (IPSS) was obtained. All the patients underwent transabdominal ultrasound to document the prostate size and intravesical prostatic protrusion. In Group 1 patients were offered at least a trial to self-void after removal of Foley Catheter before posting for surgery and the duration of Foley catheterization and the previous number of TWOC attempts were documented.

Patients with a positive urine culture were treated with appropriate antibiotics for seven days, following which urine culture was repeated. Patients with repeat culture positivity were admitted and treated with an injectable antibiotic for three days prior to surgery. All the surgeries were done under spinal anesthesia by four experienced urologists. All patients received prophylactic Ceftriaxone 1gm at the time of anesthesia. TURP was performed using 26 French rotating continuous flow monopolar resectoscopes with standard monopolar loop (Karl Storz, Tuttlingen, Germany) and generator Force FX (Medtronic, Minneapolis, MN, USA) set at 130 Watt pure cut and 80 Watt spray coagulation mode. Glycine (1.5%) was used as the irrigation fluid. At the completion of TURP, 22 French three-way Foley catheter was inserted per urethra and inflated with 30 mL distilled water. Continuous bladder irrigation was commenced with a normal saline solution which was maintained for 24 hours. Resected tissue was weighed using a digital weighing machine and was submitted for histopathological assessment. The catheter was removed on the second postoperative day. Patients were discharged on the same day once they voided twice after removal of the catheters. On failure of this trial the patients were recatheterized and discharged on the same day with advice to follow up after a week for a trial off the catheter.

Perioperative variables such as duration of surgery, the weight of resected tissue, hemoglobin change, duration of post-operative Foley catheterization, duration of hospital stay, the need for post-operative Foley recatheterization and complications were recorded. The operating time was calculated as the time from the introduction of the resectoscope till the placement of the Foley catheter.

The proportion of prostate resected was calculated as the ratio of the volume resected and the preoperative prostate volume measured under ultrasound. Post-operative hemoglobin level was assessed at 6 hours and 24 hours after the operation. Change in hemoglobin level was obtained as the difference between its preoperative and postoperative value. Serum electrolytes were assessed immediately and 24 hours after the operation. Complications were classified as per the modified Clavien classification system.

Patients were followed up at two weeks in Urology OPD to assess for hematuria, urinary tract infection and review of histopathology report. Qmax, PVR volume, IPSS and complications were reassessed at three months follow up.

Data analysis was done using the Statistical Package for Social Sciences Windows version 23 (SPSS Inc, Chicago, IL, USA). Patient demographic and clinical characteristics were compared between the two groups. Baseline characteristics were compared using the Chi-square test for categorical variables and the Student's t-test/Mann Whitney U test for continuous data. Two-tailed tests were used. One-way ANOVA and Kruskal Wallis test were used to compare continuous variables in more than two groups as appropriate. A p-value < 0.05 was considered statistically significant.

## Results

**Figure 1:**
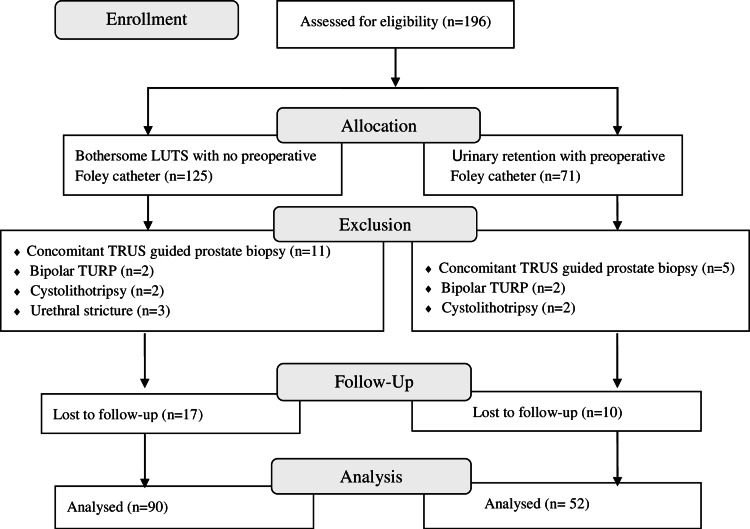
Flow chart of the study TURP: Transurethral resection of prostate; LUTS: Lower urinary tract symptoms; TRUS: Transrectal ultrasound

A total of 196 patients underwent TURP during the study period. Fifty-four patients were excluded from the study and 142 patients were included in the final analysis (Figure 1).

The patients in Group 1 were older than those in Group 2 (70.87 ± 9.74 vs 66.39 ± 9.04 years). PSA level was higher in Group 1 (3.87 ± 2.07 ng/mL) than Group 2 (2.77 ± 1.61 ng/mL), p<0.001. The patients without Foley catheters had a higher prevalence of comorbidities (51.1%) as compared to those with it (28.8%), p=0.01. Diabetes mellitus, hypertension and chronic airway disease were the common comorbidities in both groups. The mean duration of preoperative Foley catheterization in the catheterized group was 8.25 weeks (range 1 to 32 weeks) (Table 1). The mean prostate volume was higher in Group 1 (53.85 ± 15.90 mL) than in Group 2 (49.38 ± 16.19 mL); however, it was not statistically significant (p=0.120) (Table 1). Preoperative urine culture at any time prior to surgery was positive in 18 (34.6%) and 4 (4.4%) patients in Group 1 and Group 2, respectively (Table 1).

**Table 1:** Preoperative characteristics of the study population Data presented as mean ± standard deviation or number (%)  Group 1: with preoperative Foley catheter; Group 2: without Foley catheter. *Student's t-test    **Chi-square test  ***Mann-Whitney U test ASA: American Society of Anesthesiologists; BMI: Body mass index; COAD: Chronic obstructive airway disease; c/s: culture and sensitivity; DM: Diabetes mellitus; HTN: Hypertension; IVPP: Intravesical prostatic protrusion; IPSS: International Prostate Symptom Score; mL: milliliter; mm: millimeter; mths: months; NA: Not applicable; PSA: Prostate-specific antigen; PVR: Post-void residual; Q1: First quartile; Q3: Third quartile; Qmax: maximum urinary flow rate; QOL: Quality of life; yrs: years.

Variables	Group1 (n=52)	Group 2 (n=90)	P-value
Age (yrs)	70.87 ± 9.74	66.39 ± 9.04	0.007^*^
BMI (kg/m^2^)	26.55 ± 3.29	26.74 ± 2.43	0.695
ASA Score			0.010^**^
I	37 (71.1%)	41 (45.5%)	
II	15 (28.8%)	47 (52.2%)	
III	0 (0%)	2 (2.2%)	
Comorbidities			
Yes, n (%)	15 (28.8%)	46 (51.1%)	0.010^**^
DM (n)	3	10	
COAD (n)	4	13	
HTN (n)	6	20	
Others (n)	2	3	
Duration of symptoms (months) median (Q1, Q3)	12 (6, 24)	18 (10, 36)	0.207^***^
IPSS (Total)	NA	25.58 ± 5.69	NA
Storage subscore	NA	8.55 ± 3.53	NA
Voiding subscore	NA	17.02 ± 3.17	NA
QOL score	NA	5.72 ± 0.56	NA
PSA (ng/mL)	3.87 ± 2.07	2.77 ± 1.61	0.001^*^
Prostate volume (cm^3^)	53.85 ± 15.90	49.38 ± 16.19	0.113
IVPP (mm)	15.45 ± 4.80	13.69 ± 6.73	0.099
PVR (mL) median (Q1, Q3)	NA	79.50 (40, 136)	NA
Qmax (mL/sec)	NA	9.20 ± 3.11	NA
Urine c/s positive n (%)	18 (34.61)	4 (4.44)	<0.001^**^

The mean operating duration was longer in Group 1 (54.52 ± 16.48 mins) than in Group 2 (44.52 ± 15.24 mins) with a higher volume of prostatic tissue resected (27.87 ± 10.90 vs 23.14 ± 8.67 gm) (Table 2). However, the proportion of prostate volume resected was similar between the groups. There was no difference in the hemoglobin level change and duration of postoperative Foley catheterization (Table 2). Patients without catheters had a shorter duration of hospital stay (3.43 ± 0.76 days) than those with catheters (3.43 ± 0.76 days), p=0.048. Three patients in each group required reinsertion of Foley catheter because of failure to void after catheter removal on the second postoperative day (Table 2).

**Table 2:** Comparison of perioperative variables between the groups Data presented as mean ± standard deviation and number (percentage). *Student's t-test    **Chi-square test gm: gram; Hb: Hemoglobin; min: minutes; %: percent.

Variables	Group 1 (n=52)	Group 2 (n=90)	P-value
Operation duration (min)	54.52 ± 16.48	44.52 ± 15.24	< 0.001^*^
Need of active urethral dilatation	13 (25%)	37 (41.1%)	0.053^**^
Resection weight (gm)	27.87 ± 10.90	23.14 ± 8.67	0.005^*^
Proportion resected (%)	51.27 ± 11.92	47.59 ± 11.61	0.074
Hb change (g/dL)	1.40 ± 1.12	1.76 ± 1.24	0.093
Catheter time (days)	2.46 ± 1.09	2.16 ± 0.77	0.054
Hospital stays (days)	3.76 ± 1.24	3.43 ± 0.76	0.048^*^
Catheter free at discharge n (%)	49 (90.3%)	87 (96.6%)	0.487^** ^

The overall complication rate was 16.1 % with no significant difference between the groups (p=0.126) including those with failure to void after catheter removal. The majority of the complications were Clavien grade I (69.5%) and Clavien grade II (17.39%). The overall rate of recatheterization at discharge was 4.2% with 5.7% and 3.3% of patients in Group 1 and Group 2 requiring so respectively. A patient in Group 2 developed transurethral resection syndrome and he had an uneventful recovery after two days of management in the intensive care unit (Table 3). Two patients with catheters (3.8%) and three patients without catheters (3.3%) had a urethral stricture.

**Table 3:** Comparison of complications between the groups ICU: Intensive care unit; TUR: Transurethral resection; UB: Urinary bladder.

Grade	Complications	Group 1 (n=14)	Group 2 (n=9)	Management
I	Fever(n)	2	2	Antipyretic added
	Hematuria (n)	2	0	Prolonged bladder irrigation
	Catheter malfunction due to clot (n)	2	2	Bedside catheter change
	Acute retention after Foley removal (n)	3	3	Foley recatheterization
II	Post op hematuria (n)	1	0	Transfusion
	Fever with signs of bacteremia (n)	2	1	Antibiotic change
IIIa	UB clot (n)	2	0	Cystoscopy and clot evacuation
IVb	TUR syndrome (n)	0	1	Admission in ICU

Patients in Group 2 demonstrated higher post-operative Qmax than those in Group 1 (19.75 ± 3.12 mL/sec vs 16.90 ± 2.92 mL/sec, p=0.00), with no difference in PVR volume between the groups (Table 4). The subjective improvement was significantly higher in those with catheters preoperatively as demonstrated by lower mean IPSS and storage symptoms score (p=0.013) (Table 4).

**Table 4:** Comparison of outcome variables at three months follow up Data presented as mean ± standard deviation Group 1: with preoperative Foley catheter; Group 2: without Foley catheter. *Student's t-test    **Mann-Whitney U test IPSS: International Prostate Symptom Score; PVR: Post-void residual; Qmax: maximum urinary flow rate; Q1: First quartile; Q3: Third quartile; QOL: Quality of life.

Variables	Group 1 (n=52)	Group 2 (n=90)	P-value
Catheter free at follow up (%)	100	100	
Qmax (mL/sec)	16.90 ± 2.92	19.75 ±3.12	<0.001^*^
PVR (mL) median (Q1, Q3)	40 ( 20, 50)	25 (10, 50)	0.922^**^
IPSS (Total)	6.63 ± 2.68	7.46 ± 2.22	0.050
Storage subscore	2.13 ± 1.64	2.73 ± 1.35	0.021^*^
Voiding subscore	4.50 ± 1.44	4.72 ±1.36	0.363
QOL score	1.23 ± 0.42	1.44 ± 0.52	0.013^*^

There was no difference in the preoperative prostate volume and volume of prostate resected during TURP. Postoperative hemoglobin change, duration of Foley catheterization postoperatively, IPSS score and Q max at three months were similar between the groups (Table 5). Among patients with preoperative Foley catheters for more than 12 weeks, complications occurred in three patients and two patients required recatheterization after failure to void postoperatively (Table 5).

**Table 5:** Comparison between patients with various duration of Foley catheterization preoperatively Data presented as mean ± standard deviation and numbers *One-way ANOVA test  **Kruskal Wallis test  ***Chi-square test gm: gram; g/dL: gram/deciliter; Hb: Hemoglobin; IVPP: Intravesical prostatic protrusion; IPSS: International Prostate Symptom Score; mL: mililiter; PSA: Prostate-specific antigen; PVR: Post-void residual; Q1: First quartile; Q3: Third quartile; Qmax: maximum urinary flow rate; QOL: Quality of life.

	Duration of preoperative perurethral Foley catheterization				
Variables	<4 weeks (n=19)	4-8 weeks (n=15)	8-12 weeks (n=9)	> 12 weeks (n=9)	P-value
Prostate volume (mL)	50.31 ± 13.79	57.43 ± 20.00	55.45 ± 14.79	53.76 ± 14.46	0.315
Resected Prostate volume (gm)	25.15 ± 9.79	28.10 ± 12.65	30.16 ±10.39	30.94 ± 10.94	0.008^*^
Resected proportion (%)	49.72 ± 12.35	48.24 ± 10.37	54.06 ± 11.79	56.78 ± 13.02	0.301
Hb change (g/dL)	1.28 ± 1.20	1.62 ± 1.20	1.65 ± 0.99	1.04 ± 0.99	0.561
Postoperative Foley catheter duration (days)	2.58 ± 1.30	2.33 ± 0.61	2.00 ± 0.00	2.89 ± 1.61	0.340
PVR at three months (mL) median (Q1, Q3)	25 (10, 50)	45 (30, 50)	28 (10, 40)	45 (10, 55)	0.112^**^
QOL score at three months	1.36 ± 0.49	1.26 ± 0.45	1.11± 0.33	1.00 ± 0.00	0.139
IPSS at three months	6.16 ± 2.93	6.87 ± 2.85	7.11 ± 2.20	6.78 ± 2.58	0.806
Qmax at three months (mL/sec)	16.77 ± 2.46	18.13 ± 3.50	16.55 ± 2.00	15.44 ± 3.08	0.166
Complication (n)	7	2	2	3	0.449 ^***^
Foley catheter at discharge (n)	1	0	0	2	0.114^***^

## Discussion

A significant number of patients in developing countries still undergo BPH-related surgery for AUR after the failure of TWOC, which is in contrast to western countries where bothersome LUTS accounts for the majority of the cases [16,17]. Despite the availability of newer modalities, the availability and cost issues still make TURP the most frequently performed procedure [10,18]. Our study did not find differences in postoperative complication and recatheterization rates between those with and without preoperative Foley catheterization. Although the Qmax was lower in those with the preoperative catheters, they demonstrated higher subjective satisfaction reflected by their lower postoperative IPSS and QOL scores.

Increased adverse perioperative outcomes had been documented in patients in men with urinary retention [11,12,19,20]. In our study, the overall complication rate was 16.1% with the majority being Clavien I (69.5%) with a higher complication rate in Group 1 than in Group 2 (26.9% vs 10%). This was similar to that reported in other studies [20,21]. The higher rate of UTI in our study may be due to the increased prevalence of preoperative urine infection in patients with AUR. Our findings are in agreement with other studies reporting a higher rate of infection in those with urinary retention [11,19].

Prolonged duration of surgery and higher volume of resection has been shown to increase complications [19]. In our study, the operation duration was longer with the higher resected volume of the prostate in those with preoperative catheterization. However, there was no significant difference with regard to complications higher than Clavien II. The improvement in complication rate in recent studies can be attributed to the advancements in surgical technique and technology related to the instrument's design [13,15,22].

A transfusion rate of 2% to 5% has been reported in various studies [22-25]. Chen et al. reported a higher transfusion rate (3.2% vs 1.5%) and hematuria (8.1% vs 7.4%) in patients with AUR than those without AUR [11]. In our study, one patient (1.9%) required transfusion and two patients (3.8%) required cystoscopy and clot evacuation in a preoperatively catheterized group of patients while none of the patients without a catheter had hematuria or required transfusion. Sagen et al. also reported higher blood loss in patients with AUR [25]. Catheter-induced cystitis and prostatitis with fragile vessels after prostatic infarction have been implicated in increased blood loss in patients with per-urethral catheters [12,19,20].

In our study, TUR syndrome was noted in one patient (0.7%) and he recovered uneventfully after two days of management in the intensive care unit. Our findings are in accordance with the review which reported 0%-1% incidence of transurethral resection syndrome [24].

A higher proportion of men failing to void after TURP has been documented in those with preoperative urinary catheters [11,12,17,19,23]. In our study, recatheterization at the time of discharge was required in 5.7 % and 3.3 % of patients with and without preoperative catheterization respectively at the time of discharge. All these patients were catheter-free at one week of surgery. A higher recatheterization rate was reported in patients with catheters preoperatively with 23% of the patients with retention requiring catheters in contrast to only 8% of those patients without preoperative retention [25]. Chen et al. also reported a 13.8% recatheterization rate in patients with AUR whereas none of the patients without AUR required so [11]. Similar to our study Johnsen et al. did not find a difference in recatheterization rate regardless of preoperative retention status [14].

In our study, the patients with retention were older with larger prostate and had longer operative duration and greater volume of prostate resected. The mean PSA was higher in a catheterized group (3.87 ± 2.07 vs 2.77 ±161 ng/mL). Similar findings were documented by Sagen et al. [25]. The higher prostate volume and the Foley catheter-induced prostatic inflammation may have led to raised PSA in this group.

The mean preoperative IPSS and QOL scores in the non-catheterized group were 25.70 and 5.72, respectively, which was similar to the findings by Luitel et al. but higher than reported by Choi et al. [15,26]. The higher IPSS score may be due to the delayed presentation of our patients to seek care after the symptoms were significantly bothersome. In this study, the patients with preoperative catheterization had better subjective symptoms scores as reflected in the lower postoperative IPSS and QOL scores. These findings are similar to the study Sagen et al. [25] where patients with AUR had lower IPSS and QOL scores than those without AUR. In contrast to our findings, Chuang et al. [27] did not find a significant difference in the scores between those with and without retention. Getting rid of the urethral catheters may have also contributed to better QOL in those with catheters preoperatively.

Patients with catheters had significantly lower maximum flow rates postoperatively than those without. The cause of AUR in these patients may be the combination of BEP and some degree of detrusor insufficiency which may have affected the Qmax. A similar reduction in flow rate in patients with AUR was reported by Choi et al. [13]. In contrast, Sagen et al. did not find a difference in maximum flow rate between the groups [25].

Patients in our part of the world present late due to financial reasons, a misconception that it is the normal aging process, fear of surgery and need to travel a greater distance to seek a cure and thus need to have a catheter for a longer duration preoperatively [5,15]. This may be the reason for the significant number of patients having catheters for more than four weeks preoperatively. Prolonged urinary catheterization has been associated with a significantly higher rate of adverse events such as hematuria, and infection, especially in older patients [3,12,28]. We did not find a difference in the mean change in hemoglobin, resected prostate volume and, duration of Foley catheter postoperatively with regards to the various duration of preoperative catheterization. Similarly, Qmax, IPSS and QOL scores at three months were also similar in those subgroups. Increased complications were observed in patients with preoperative Foley catheterization for more than 12 weeks together with the non-significant increased need for recatheterization before discharge. This may be due to the morbidity associated with prolonged catheterization. Similar findings were noted in a study by Das et al. who also failed to find differences in recatheterization rate even in patients with a longer duration of preoperative catheterization [29].

The loss to follow up of a significant number of patients in this sample size makes it the major limitation of the study. Secondly, the duration of follow-up of only three months duration was short to assess long-term post-operative voiding outcomes and complications. The study being a single-centered study limits the generalizability of the results. Transabdominal ultrasound was used to estimate prostate volume instead of transrectal ultrasound which would have provided a better estimation of the prostate volume.

## Conclusions

Our study demonstrated similar postoperative voiding outcomes in patients with preoperative per-urethral catheterization with comparable complication rates to those without catheters. Patients with catheters had better postoperative subjective symptom scores in spite of having a lower postoperative maximum flow rate than those without it. There was no significant difference in complication rate even in those with prolonged catheterization.

These findings have implications for urologists and patients as a significant number of patients still undergo surgery for BPH after suffering an episode of AUR and need to be on an indwelling catheter for a longer duration due to various causes in our part of the world. Previously, these patients were reluctant to undergo surgery as they were counseled regarding inferior postoperative improvements in voiding parameters. The findings of our study may help in counseling these patients as they can expect similar improvement in voiding parameters as those without catheters. Further clinical studies with large sample size and longer follow-up period are warranted to validate these findings.
